# Influence of the Initial Prestress Level on the Reliability Index of Geiger Dome

**DOI:** 10.3390/ma18235291

**Published:** 2025-11-24

**Authors:** Paulina Obara, Urszula Radoń, Paweł Zabojszcza

**Affiliations:** Faculty of Civil Engineering and Architecture, Kielce University of Technology, al. Tysiąclecia Państwa Poskiego 7, 25-314 Kielce, Poland; paula@tu.kielce.pl (P.O.); zmbur@tu.kielce.pl (U.R.)

**Keywords:** reliability index, tensegrity systems, cable-strut domes, initial prestress level

## Abstract

This paper presents a reliability analysis of four distinct design solutions, known as Geiger domes. These domes possess two intrinsic characteristics of tensegrity structures: infinitesimal mechanisms and self-stress states. In the absence of self-stress (initial prestress), Geiger domes are inherently unstable; stabilization is achieved only through the introduction of initial prestressing forces. The reliability analysis is carried out using a system-based approach and focuses on domes with six load-bearing girders. Two girder geometries are examined: one with a closed upper section and the other with an open upper section. The primary aim of this study is to identify a design solution that is more sensitive to the influence of initial prestress on structural reliability. Accordingly, the reliability index is evaluated as a function of the initial prestress level. Within the system-based framework, the reliability assessment of tensegrity structures is conducted in three stages. The first stage involves identifying self-stress states and infinitesimal mechanisms. The second stage concerns the formulation of reliability models, while the third stage evaluates the effect of initial prestress on the reliability index. Research has shown that the best solution is the original Geiger dome.

## 1. Introduction

The first tensegrity cable and strut dome, proposed and patented in 1988, was the Geiger dome [1]. The main principle of this type of dome is that stress is transferred through the roof structure by tensioned cables and discontinuous compression struts. The original structure consisted of radial trusses, with tensed and compressed elements. A characteristic feature of this type of roof is its low profile, which reduces wind uplift, uneven snow deposition, and wear of roofing material. One of the main advantages of this structure is its weight per square meter, which remains constant as the span increases. This solution was used on the roof of the Olympic Gymnastics Hall in Seoul [2]. A characteristic feature of Geiger domes is the system of internal forces that keeps the structural components in stable equilibrium. This system is called the self-stress state or initial prestress. In the absence of the initial prestress, the domes are unstable, i.e., geometrically variable (infinitesimal mechanisms occur). Stabilization only occurs when the initial prestress is introduced. In addition, changes in the level of initial prestress affect the behavior of the domes.

After its first appearance, the Geiger dome was the main subject of many scientific works. The first modifications of the Geiger dome led to adjusting the cable layout and providing additional cables. The modification was presented by Kim et al. [3] and relied on the additional intersecting bracing cables. In later works, the crossing cables were removed and only the additional circumferential cables were left [4]. The original geometry of the Geiger dome motivated other scientists to create new shapes (generate new topologies) [5,6,7], and present new form-finding [8,9,10] and optimization methods [6,11,12]. New cable dome types appeared based on a Geiger dome patent [13,14]. As part of an experimental study, different construction methods [15] and shape-forming processes [16,17] were presented. The new topology aimed to achieve the desired performance criteria and enable control of parameters such as stiffness [18] and stability performance [19,20,21]. The static analysis of Geiger domes includes the influence of the initial prestress level on the structure’s response. The literature review shows works that study the influence of the initial prestress on the displacements [5,18,22], effort and stiffness of the structure [22,23], and frequencies [23,24]. The dynamic stability of Geiger domes is also considered [23,25]. On the other hand, the reliability analysis of the Geiger domes is a subject that is still understudied. At this point, it should be noted that the reliability assessment is a key issue in the design of structures.

One of the pioneering and most influential algorithms for evaluating the reliability of structures subjected to combined loading conditions was proposed by Rackwitz and Flessler in 1978 [26]. This development represented an important step forward in the formalization of structural reliability analysis. A particularly significant breakthrough in this field was the introduction of the Hasofer–Lind reliability index, which became a cornerstone of modern reliability theory [27]. Unlike the previously used Cornell index, whose major limitation was its lack of invariance under linear transformations, the Hasofer–Lind index provided a consistent and robust measure of reliability [28]. The adoption of this index laid the groundwork for more efficient and systematic implementation of both the First-Order and Second-Order Reliability Methods (FORM and SORM), thereby substantially enhancing the accuracy and computational effectiveness of structural reliability assessments [29,30,31].

A modern approach to estimating failure probability involves the Stochastic Finite Element Method (SFEM). An interesting application of SFEM to steel lattice towers is presented in [32,33]. Another important trend is the use of Response Surface Methods (RSM) [34], with notable applications to the design of composite panels discussed in [35,36]. Furthermore, the Monte Carlo Method has been effectively combined with artificial neural networks to enhance computational efficiency [37,38].

Most of the referenced studies focus on a single limit state. A different perspective is provided by system reliability analysis, which allows for the simultaneous consideration of multiple limit state functions. However, in such analyses, the large number of potential failure modes significantly increases the computational cost. To address this challenge, various redundancy strategies have been proposed. Kim et al. introduced a selective searching technique [39], while Safari investigated redundancy strategies for multi-objective reliability optimization [40]. An innovative approach is presented in [41], where the authors employ a K-mixed redundancy strategy. They first develop the mathematical formulation for calculating the reliability of the K-mixed strategy, and subsequently evaluate its effectiveness and efficiency across various test cases, including a well-known benchmark problem.

Reliability methods have also proven valuable in addressing diverse engineering problems, particularly in civil engineering. Siacara et al. demonstrated how accurate and efficient reliability analyses of geotechnical installations can be performed by directly coupling geotechnical software with a reliability solver [42]. The work [43] presents a comprehensive framework for applying structural reliability and sensitivity analyses in engineering practice. The authors describe the use of both FORM and Monte Carlo simulation for failure probability assessment, as well as local and global sensitivity analyses. Examples include the use of derivative-based sensitivity measures for reliability indices and Sobol indices for global sensitivity within Monte Carlo simulations. The study [44] focuses on the optimization of a steel roof frame while explicitly accounting for the randomness of key design parameters. The authors employed geometrically nonlinear finite element analysis (FEA) combined with the First-Order Reliability Method (FORM) to compute reliability indices. They compared traditional deterministic optimization with two alternative approaches: robust optimization and reliability-based design optimization (RBDO). Ontiveros-Pérez and Miguel proposed a methodology for the reliability-based optimal design of multiple tuned mass dampers for buildings in seismic regions [45]. In [46], an innovative approach to topology optimization based on thermoelastic-plastic reliability was considered. The authors presented a modern Bidirectional Evolutionary Structural Optimization (BESO) method that combines the thermoelastic-plastic finite element method with stochastic reliability constraints. This solution allows for the development of structural designs under thermal and mechanical loading. Reliability-based optimization issues are also used in the design of geotechnical structures, such as piles. In [47], the ultimate load-bearing capacity of laterally loaded piles was analyzed. For this purpose, a nonlinear mathematical programming procedure was developed that utilized a probabilistic constraint on the value of the residual force supplementary strain energy to determine the safe load capacity of a long laterally loaded pile.

The reliability of steel structures under fire conditions is discussed in [48,49], while [50,51] present new methods for identifying cut sets in system reliability analysis.

The overall level of safety is largely determined by the interaction of the structural elements in the load-bearing system and the safety of the individual elements. There are four groups of reliability assessment methods. In each of these methods, a reliability index is a common measure of the overall reliability of the structure. The most comprehensive reliability analysis is the system approach [52,53,54]. This method allows a distinction to be made between the two relevant concepts that are closely related and often equated, namely the reliability model and structural safety.

In the paper, reliability analysis of four different design solutions called Geiger domes is carried out. The analysis is performed using the system approach. The analysis involved domes with six supporting girders, in two variants of girder geometry: a closed upper section (type A) and an open upper section (type B). In addition, two designs are taken into account, i.e., regular (according to Geiger patent) and modified (with additional cables). The paper compares four different Geiger dome designs solutions, i.e., regular dome type A [8], regular dome type B [55], modified dome type A [56], and modified dome type B [57].

This article addresses the question of which type of structural solution is more sensitive to the effect of prestressing on reliability. For this purpose, the reliability index as a function of the initial prestress is determined. Reliability analysis in the system approach of tensegrity structures is a three-stage process. The first stage involves identifying residual stress states and infinitesimal mechanisms. The second stage focuses on defining reliability models, while the third stage involves determining the effect of prestressing on the reliability index.

The original contribution of this study lies in the application of a system-based reliability analysis to tensegrity structures. The combination of these two research areas—reliability system theory and the mechanics of tensegrity structures—represents an innovative step toward the quantitative assessment of safety and durability in such systems.

Previous studies on tensegrity structures have primarily focused on their geometric behavior, stiffness, or optimization of prestressing forces, while probabilistic and reliability aspects have largely been neglected. In this work, for the first time, the Geiger dome is treated as a system with a defined logical structure (a series system), which enables a quantitative evaluation of the influence of initial prestressing on the probability of failure and the corresponding reliability index.

This approach not only provides a more realistic representation of structural behavior under random variations in loads and material parameters but also forms a foundation for the further development of reliability assessment methods for other types of tensegrity structures.

## 2. Materials and Methods

The fundamental principles of structural design with respect to reliability criteria are specified in [58]. Structural reliability—defined as the ability of a structure to perform its intended functions without failure throughout its design life—constitutes a key quality criterion and a primary requirement for all load-bearing systems. For standard building structures, the recommended design life is 50 years. Depending on the type of structure and the potential consequences of its failure, three reliability classes (RCX) corresponding to three consequence classes (CCX) are adopted. In this study, the authors assumed that tensegrity domes correspond to reliability class RC2.

Structural safety is characterized by two global parameters, i.e., the effect of actions $N_{P}$ and the load-bearing capacity $N_{Rd}$. A fundamental measure of structural failure is the probability of failure $P_{f}$ defined as the probability of events ω in which the load-bearing capacity of an element is exceeded. For rationally designed structures, $P_{f}$ is typically very low, ranging from approximately 10^−4^ to 10^−7^. However, using this measure directly would require extremely high computational precision and would pose significant challenges for practical interpretation and presentation. Therefore, a more practical and widely adopted measure is a reliability index β, commonly used in both standards and scientific literature. Relationship between the failure probability $P_{f}$ and reliability index β is shown in Table 1. The procedure for determining these quantities at both the element and system levels is described in detail later in this paper. For the ultimate limit state and reliability class RC2, the required minimum reliability index is β=3.8 (Table 2). The failure probability $P_{f}$ and the reliability index β will be calculated using the First-Order Reliability Method (FORM), an approximation technique widely applied in structural reliability analysis.

The article focuses on determining the reliability index of Geiger domes. These structures are characterized by the presence of two inherent features of tensegrity structures: an infinitesimal mechanism and a residual stress state. Geiger domes are unstable when no residual stress (initial prestress) is present. The stabilization occurs only when the initial prestress forces are introduced. This study examines the effect of prestress on the reliability index. In the first stage, the feasible prestressing range is determined by calculating the minimum and maximum prestress level. Subsequently, for selected prestressing force values, the nonlinear equilibrium equations are solved. This procedure yields the normal forces, the stress state of each member of the dome, and the corresponding reliability index. The obtained reliability index values are then presented as a function of the initial prestress level.

### 2.1. Minimum and Maximum Prestressing Forces

The Geiger dome is modeled using the finite element formalism [60]. The components of dome are modeled as the space finite tensegrity element [23]. The n-element structure (e=1,2,…,n) with m degrees of freedom described by a displacement vector $q\in R^{m\times 1}$ is considered. The components of structure are modeled as the space finite tensegrity elements e of Young’s modulus $E_{i}$, density $\rho_{i}$, cross-sectional area $A_{i}$, and length $L_{i}$. The structure is loaded by forces applied at the nodes $P\in R^{m\times 1}$. Complete reliability analysis of Geiger dome is a two-stage process. The first stage includes the identification of infinitesimal mechanism and self-stress state $y_{s}$. Both features depend only on the elongation matrix $B\in R^{n\times m}$ [23]. Zero eigenvalues of the matrix $BB^{T}\in R^{n\times n}$ are responsible on existing the self-stress states, while zero eigenvalues of the matrix $B^{T}B\in R^{m\times m}$ on existing the infinitesimal mechanisms. The self-stress state is considered as an eigenvector $y_{S}$ related to zero eigenvalue of the matrix $BB^{T}$. The eigenvector $y_{S}$ allows us to define the self-equilibrated forces in elements:

(1) $$S=y_{s}S\;\in R^{n\times 1},$$

where S is the initial prestress level.

The range of initial prestress levels in each structure is unique and depends on the design assumptions and external loads:

- The minimum prestress level $S_{min}$ is related to the appropriate distribution of normal forces in the structural elements, i.e., the cables must be in tension and the braces must be in compression. Changes in the distribution of normal forces, which may be caused by external loading, can be corrected by introducing an appropriate initial prestress level.
- The maximum prestress level $S_{max}$ is related to the load-bearing capacity of the most-stressed elements, i.e., on the effort of the structure:
  (2) $$W_{\max}=\frac{N_{max}}{N_{\mathrm{Rd}}}$$
  where $N_{\max}$ is the normal force in a most-stressed element.

### 2.2. Normal Forces in Elements

To calculate normal forces, a geometrically nonlinear model is used [23]. As a basis for formulating the tensegrity lattice equations, the nonlinear theory of elasticity in Total Lagrangian—TL—approach is adopted. The incremental static equilibrium equation takes the following form:

(3) $$K_{T}\left(q\right)∆q=∆P+R$$

where $R\in R^{m\times 1}$ is the residual force vector results from the aggregation (in equilibrium, it is equal zero (R=0), while in the iteration process the norm $\left\|R\right\|$ is the “distance” from the equilibrium state; the iterative process converges if $\left\|R\right\|\to 0$) and $K_{T}\left(q\right)\in R^{m\times m}$ is the tangent stiffness matrix of structure presented as follows:

(4) $$K_{T}=\left[K_{L}+K_{G}\left(S\right)+K_{GN}\left(P\right)+K_{NL}\left(q\right)\right],$$

where $K_{L}$—linear stiffness matrix, $K_{G}\left(S\right)$—geometric stiffness matrix (prestress matrix) depending on the self-equilibrated forces in elements (1), $K_{GN}\left(P\right)$—geometric stiffness matrix depending on the longitudinal forces which results from external loads, $K_{NL}\left(q\right)$—nonlinear part consists with the symmetric displacement stiffness matrix

(5) $$K_{NL}\left(q\right)=K_{u1}+K_{u2}.$$

The most important in the analysis of Geiger domes includes the stiffening of the structure under the influence of external load. The stiffness matrix $K_{GN}\left(P\right)$ is calculated in four steps:

- Step 1—determining displacements from the nonlinear system of equilibrium Equation (3), assuming $K_{GN}\left(P\right)$ equals to zero.
- Step 2—determining deformation of the element in the actual configuration:
  (6) $$\varepsilon =\frac{1}{2}\frac{{\left(L_{i}^{a}\right)}^{2}-{\left(L_{i}\right)}^{2}}{{\left(L_{i}\right)}^{2}};\;\;\;\;L_{i}^{a}=\sqrt{{\left(\Delta_{u_{2}}\right)}^{2}+{\left(\Delta_{u_{3}}\right)}^{2}+{\left(L_{i}+\Delta_{u_{1}}\right)}^{2}},$$
  where $L_{i}^{a}$ is the length of the i-th element in the actual configuration, and $\Delta_{u_{i}}=q_{i}^{2}-q_{i}^{1}(i=1,2,3)$ is the displacement increment between nodes of element.
- Step 3—determining the real normal force in the element on the basis of the Cauchy tensor:
  (7) $$N_{i}=E_{i}A_{i}\varepsilon \sqrt{1+2\varepsilon }$$
- Step 4—determining the geometric stiffness matrix $K_{GN}\left(P\right)$ depending on the longitudinal forces $N_{i}$ which results from external load P.

The nonlinear system of equations (Equation. 3) was solved using the Newton–Raphson iterative method implemented in a custom Mathematica program. The iterative process was controlled by two convergence criteria:

- The norm of the residual vector of equilibrium equations was required to fall below 10^−8^;
- The relative change in nodal displacements between successive iterations did not exceed 10^−6^.

In all analyzed cases, convergence was achieved within 4–8 iterations per load step.

Each structural element was modeled as an axially loaded, massless bar element with linear elastic behavior and constant cross-section. Cable elements were defined as tension-only, while struts carried compression only. The stiffness matrix of each element was formulated in global coordinates, allowing for geometric nonlinearity through the inclusion of updated element orientation at every iteration.

The applied load was introduced incrementally using a stepwise strategy with adaptive control: initial increments were set to 10% of the target load, and reduced automatically if convergence difficulties occurred. This approach ensured numerical stability, especially near the critical states corresponding to changes in the self-stress level.

### 2.3. Reliability Analysis of an Element

In the study, the bearing capacity of an element $N_{Rd}\left(\omega \right)$ and the effect of actions $N_{P}\left(\omega \right)$ have normal distribution. Random variables are characterized by standard deviation *σ* and the expected value μ. The reliability of an element $R\left(\omega \right)$ is defined as the probability that the bearing capacity of an element $N_{Rd}(\omega )$ will be greater than the effect of actions $N_{P}\left(\omega \right)$:

(8) $$R\left(\omega \right)=\mathrm{Probability}\left\{N_{Rd}\left(\omega \right)>N_{P}\left(\omega \right)\right\}.$$

The ultimate limit state function is defined as the difference between the bearing capacity of an element $N_{Rd}\left(\omega \right)$ and the effect of actions $N_{P}\left(\omega \right):$

(9) $$Z\left(\omega \right)=N_{Rd}\left(\omega \right)-N_{P}\left(\omega \right)$$

The random variable (9) is commonly referred to as the safety margin. Since this study assumes that both the bearing capacity and the action effects follow a normal distribution, the safety margin also exhibits a normal distribution with parameter $\mu_{Z}$ and $\sigma_{Z}$.

(10) $$\mu_{Z}=\mu_{N_{Rd}}-\mu_{N_{p}},$$

(11) $$\sigma_{Z}=\sqrt{\sigma_{N_{Rd}}^{2}+\sigma_{N_{p}}^{2}},$$

The reliability index $t_{i}$ for the i-th element can be expressed as follows:

(12) $$t_{i}=\frac{\mu_{Z_{i}}}{\sigma_{Z_{i}}}.$$

The reliability index (12) allows for the determination of the i-th element’s failure probability:

(13) $$P_{fi}=\Phi (-t_{i}),$$

where $\Phi (-t_{i})$ is the Laplace function and, consequently, the reliability of a single element:

(14) $$R_{i}=1-P_{fi},$$

The bearing capacity depends on several factors. In this study, yield strength $f_{y}$ and area of cross-section A were considered as random variables, with coefficients of variation $\nu_{fy}=0.08$ and $\nu_{A}=0.06$, respectively, in accordance with [50]. All other parameters were treated as deterministic. The coefficient of variation for bearing capacity $N_{Rd}$ is equal to

(15) $$\nu_{N_{Rd}}=\sqrt{\nu_{f_{y}}^{2}+\nu_{A}^{2}}=0.1$$

The coefficient of variation for load *P* was assumed to be $\nu_{N_{p}}=0.06$ [50], which corresponds to a standard deviation of $\sigma_{P_{k}}=0.06P$.

### 2.4. Reliability Analysis of a Structure

To analyze the reliability of a structure a systemic approach is used. In this case, it is necessary to take into account their structural layout and the interaction between structural elements. Generally, three basic models are typically used to assess system reliability, i.e., series, parallel, and mixed. A series system is one that fails if just one element stops working (becomes inoperable), because then the system becomes geometrically variable. A series system corresponds to structures, in which the load-bearing capacity of the weakest element is decisive for the load-bearing capacity of the entire structure. A characteristic feature of a series system is that as the number of its components increases, its load-bearing capacity and reliability decrease. From the perspective of reliability, the Geiger dome is modeled as a series system. This structure consists of tensioned cables and compressed struts, which together form a system of forces ensuring the geometric invariability and stability of the dome. The failure of a single element may initiate one of the infinitesimal mechanisms. Even a local failure often results in the overloading of adjacent elements and a cascading reduction in structural performance. For this reason, the reliability of the Geiger dome largely depends on the reliability of its fundamental elements. Therefore, modeling the Geiger tensegrity dome as a series system is fully justified. The reliability of structures whose configuration corresponds to a serial system is calculated using the following formula:

(16) $$R=\prod_{i=1}^{n}R_{i}.$$

Modeling the domes as pure series systems represents a conservative assumption, especially for modified configurations equipped with additional cables. The adopted approach was motivated by the primary objective of this study—to evaluate the influence of initial prestressing on the reliability index under the most unfavorable conditions.

In modified configurations, the inclusion of redundant elements may allow partial load redistribution, delaying or mitigating the onset of mechanism activation.

The future research will address more advanced system models, including parallel-series configurations, to quantify the real contribution of redundancy and load redistribution in modified tensegrity domes.

## 3. Results

**Figure 1:**
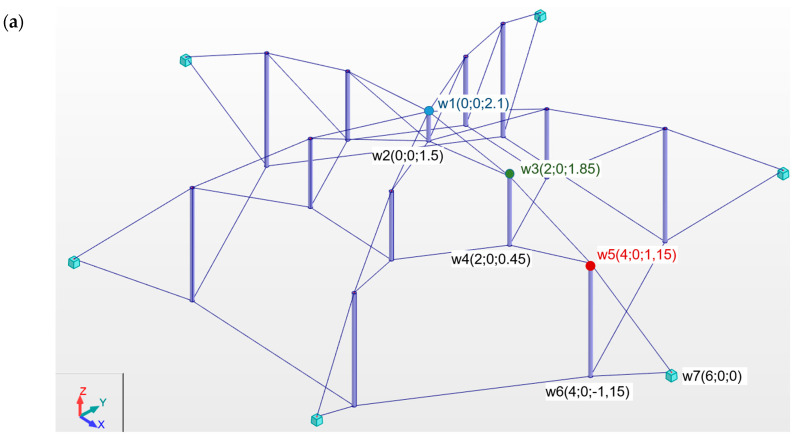

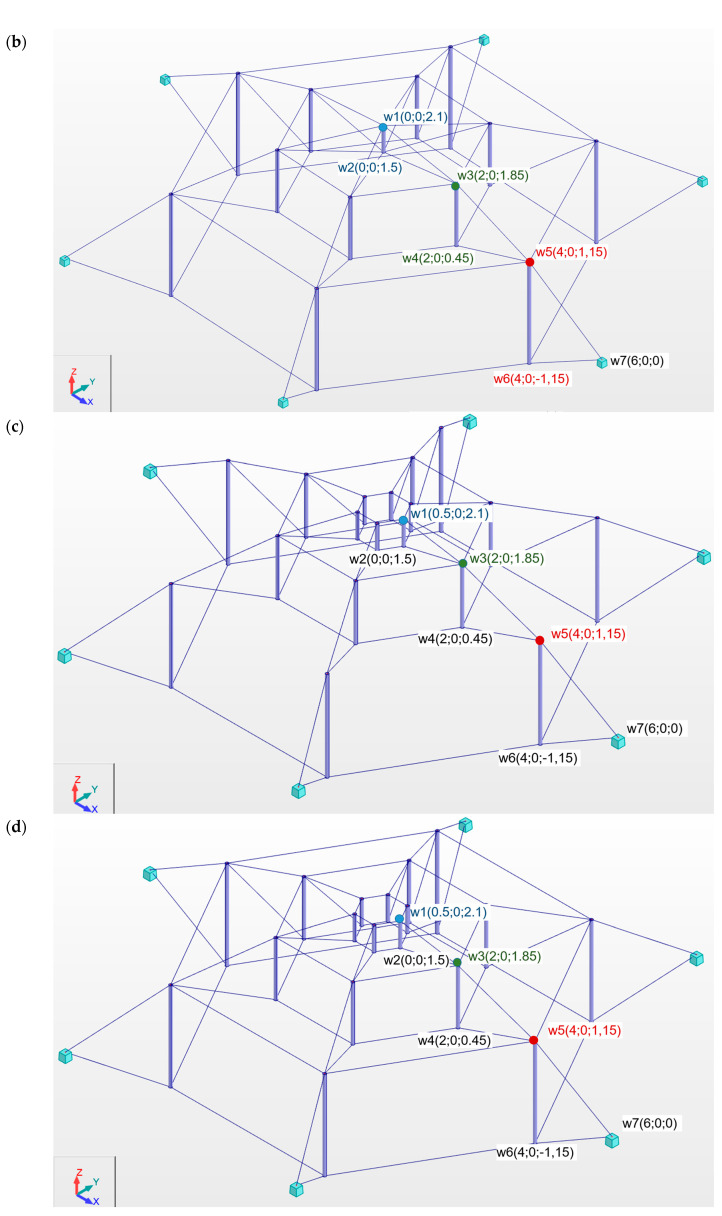
Scheme and coordinate of Geiger domes: (**a**) regular type A; (**b**) modified type A; (**c**) regular type B; (**d**) modified type B [23].

In the paper, the reliability analysis of four design solutions known from the literature of Geiger domes is performed. The domes are built with flat load-bearing girders distributed radially with respect to the axis z in the center of the span (Figure 1). Two variants of connecting in the center of the span are considered, i.e., girders connected by a strut (type A) (Figure 1a,c) and by a ring (type B) (Figure 1b,d). Two design solutions of Geiger domes were considered, i.e., a dome in accordance with the Geiger patent (standard dome) and a modification of the Geiger patent by adding additional peripheral cables (parallel 3 and parallel 5) connecting the upper nodes (modified dome).

Small steel domes were analyzed. The domes have a diameter of 12 m and a height of 3.25 m. Each dome is supported at every external node of the lower section by a pinned, non-sliding support.

The coordinates of the supporting girders are presented in Figure 1. The coordinates of the subsequent girders were determined according to polar variables as $x=r_{i}\cos\gamma$ and $y=r_{i}\sin\gamma$ (i=0,1,2,3). The following radii are assumed: $r_{0}=0.5\;m$, $r_{1}=2.0\;m$, $r_{2}=4.0\;m$, $r_{3}=6.0\;m$. The load application locations are color-coded: node 1 (blue), node 3 (green), and node 5 (red). These colors also correspond to the markings in the next figure. It is assumed that the cables in Geiger domes are made of steel S460N. The cables type A with Young modulus 210 GPa [55] are used. It was assumed that the struts were made of hot-rolled circular hollow profile (S355J2 steel) with a Young’s modulus of 210 GPa. The density of steel equals $\rho =7860\;kg/m^{3}$. The cables with diameter ϕ=20 mm with load-bearing capacity $N_{Rd}=110.2\;kN$ is taken into account. The struts rod with diameter ϕ=76.1 mm and thickness t=2.9 mm were divided into three groups according to length l and load-bearing capacity $N_{Rd}$:

- $S_{1}$ group: l=0.6 m, $N_{Rd}=224.3\;kN$,

- $S_{2}$ group: l=1.4 m and $N_{Rd}=170.5\;kN$,

- $S_{3}$ group: l=2.3 m and $N_{Rd}=107.1\;kN$.

First, four different types of domes consisting of six load-bearing girders are analyzed. Next, the influence of the number of load-bearing girders on the behavior of regular Geiger domes type B (original dome) is considered.

### 3.1. Immanent Tensegrity Features of Geiger Domes

Geiger domes are characterized by both immanent tensegrity features, i.e., infinitesimal mechanisms and self-stress states. Their number depends on the dome type. For domes with six load-bearing girders, the regular type A design has 18 mechanisms and one self-stress state; for type B, it has 31 mechanisms and one self-stress state; for the modified type A design, it has eight mechanisms and three self-stress states; and for type B, it has 21 mechanisms and three self-stress states. Generally, the modification of regular domes leads to the reduction in the number of infinitesimal mechanisms, but at the same time, the number of self-stress states increases. Unfortunately, none of them identifies the type of elements correctly. The superimposed self-stress state is needed. The normalized values on the self-stress forces $y_{S}$ for domes consisting of 6 load-bearing girders are shown in Table 3.

It is necessary to note that for ordinary Geiger domes it is possible to derive formulas for self-equilibrium forces (state of residual stress). These formulas depend on the type of load-bearing girders, the angle of inclination of the diagonal cables of girder $\alpha_{i}$, and additionally on the angle γ (2γ is the angle between circumferential cables). The case of regular Geiger domes type A:

(17) $$N_{1}=const.;i\in N_{+}$$

struts:

(18) $$N_{S1}=-6\;N_{1}\sin\left(\alpha_{1}\right)\;N_{S_{i+1}}={-N}_{2(i+1)}\sin\left(\alpha_{2\left(i+1\right)}\right)$$

diagonal cables:

(19) $$N_{2i}=N_{2i-1}\frac{\sin\left(\alpha_{2i-1}\right)}{\sin\left(\alpha_{2i}\right)}\;N_{2i+1}=\frac{N_{2i-1}\cos\left(\alpha_{2i-1}\right)+N_{2i}\cos\left(\alpha_{2i}\right)}{\cos\left(\alpha_{2i+1}\right)}\;$$

circumferential cables:

(20) $$N_{C_{2(i+1)}}={0.5N}_{2(i+1)}\frac{\cos\left(\alpha_{2\left(i+1\right)}\right)}{cos\left(\gamma \right)}$$

The case of regular Geiger domes type B:

$$N_{1}=const.;i\in N_{+}$$

struts:

(21) $$N_{S_{1}}={-N}_{1}\sin\left(\alpha_{1}\right)$$

diagonal cables:

(22) $$N_{2i}=N_{2i-1}\frac{\sin\left(\alpha_{2i-1}\right)}{\sin\left(\alpha_{2i}\right)}\;N_{2i+1}=\frac{N_{2i-1}\cos\left(\alpha_{2i-1}\right)+N_{2i}\cos\left(\alpha_{2i}\right)}{\cos\left(\alpha_{2i+1}\right)}\;$$

circumferential cables:

(23) $$N_{C_{1}}={0.5N}_{1}\frac{\cos\left(\alpha_{1}\right)}{cos\left(\gamma \right)}\;N_{C_{2}}={0.5N}_{2}\frac{\cos\left(\alpha_{2}\right)}{cos\left(\gamma \right)}$$

### 3.2. Reliability Analysis of Geiger Domes Consisting of 6 Load-Bearing Girders

First, the influence of design solution on reliability of structures is analyzed. The main purpose is to answer the question of which type of design solution is more sensitive to impact of the initial prestress on reliability. For this purpose, the reliability index β as a function of the initial prestress is determined. The analysis is cognitive in nature. The four different types of domes consisting of six load-bearing girders (Figure 1) are taken into account. A force of *P* = 5 kN was applied to the structure at one node in the z direction. This assumed load is sufficient to assess the behavior of the domes under extreme loads. For comparison purposes, the load was applied sequentially to nodes 1 (blue), 3 (green), and 5 (red).

The minimum prestress level $S_{min}$ is calculated individually for each dome for each variant of load applications (Table 4). In order to demonstrate the impact of load value, the minimum prestress level for force P=1 kN is calculated. As can be seen, only for the original dome (regular Geiger dome type B) does the minimum prestress level not depend on both position and value of external load. The value $S_{min}=2\;kN$ ensures the appropriate identification of the element type and provides the positive define matrix.

In turn, the same maximum prestress level $S_{max}=50\;kN$ was adopted to compare the behavior of all domes. This value corresponds to the maximum effort of the structure (2) equal to $W_{max}=0.93$.

Value of the minimum prestress level $S_{min}$ is related to the appropriate distribution of longitudinal forces in the elements of the structure caused by external loads. This depends on both value and position of load. Assuming a minimum prestress level of $S_{min}=0$ means that the external load causes an appropriate distribution of longitudinal forces in the elements of the structure, i.e., the cables are tensioned and the struts are compressed.

Modification of Geiger patent caused totally different distribution of longitudinal forces in the elements. This distribution mostly depends on the value and position of load. We examine the equilibrium in a deformed configuration.

**Figure 2:**
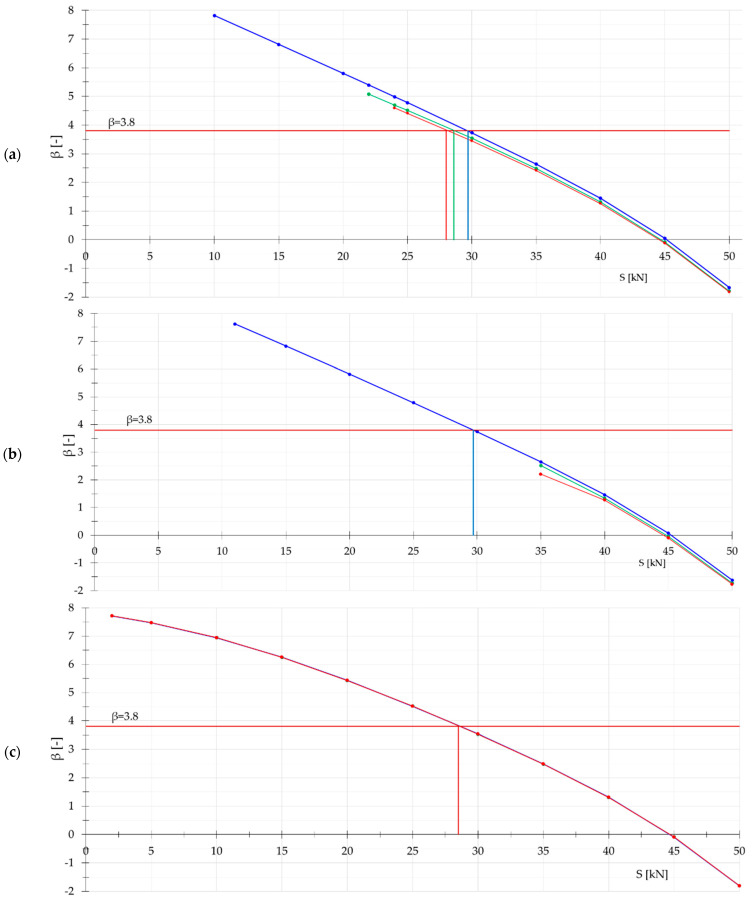

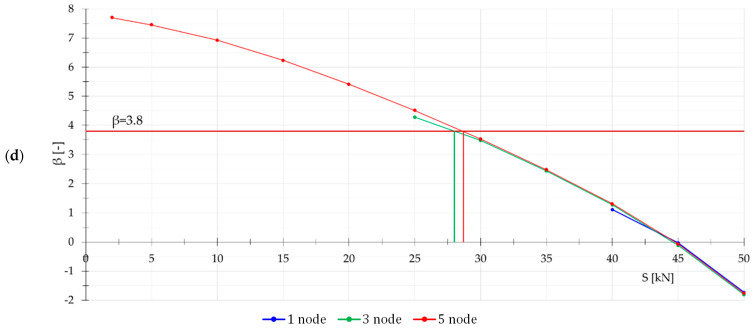
Influence of the initial prestress level S on the reliability index β for Geiger domes: (**a**) regular type A; (**b**) modified type A; (**c**) regular type B; (**d**) modified type B.

Next, the reliability index β depending on the initial prestress S is calculated. Results only for external load P=1 kN are presented in Figure 2. In the case of the original dome (Figure 2a,c), the value of reliability index does not depend on position of load (the values overlap). For the rest of domes, the situation is different; everything depends on $S_{min}$. Additionally, in the case of modified domes (Figure 2b,d), if the load is added at 5th node, the reliability index β for $S_{min}$ is significantly lower than that given in the reference. The reference point of reliability index for RC2 class structures should be greater than 3.8. For all domes, the referenced value of β corresponds to the initial prestress about S=30 kN. This means that $S_{max}$ should be calculated for effort of structure lower than $W_{max}\leq 0.6$.

### 3.3. Reliability Analysis of Regular Geiger Domes Type B Consisting of i-th Load-Bearing Girders

**Figure 3:**
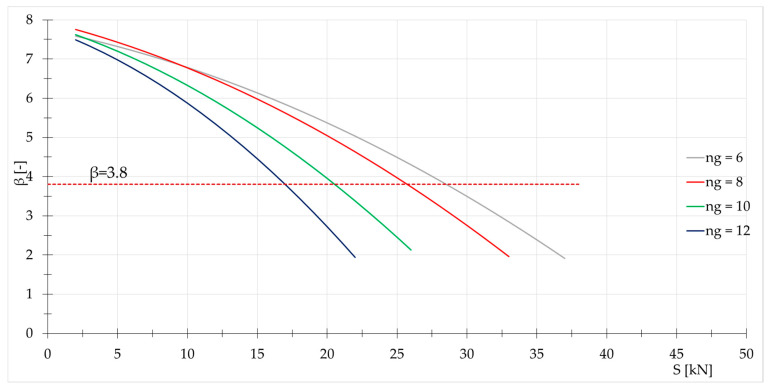
Influence of the initial prestress level S on the reliability index β.

The best solution resulting from the reliability analysis is the original Geiger dome (regular Geiger dome type B). Therefore, for this structure, the influence of the number of load-bearing girders ng on the behavior is considered. The structures with 6, 8, 10, and 12 girders are compared. The results for domes loaded by force P=5 kN applied at 1st node are shown in Figure 3. As can be seen, the value of $S_{max}$ decreases as the number of load-bearing girders increases.

It should be noted that in each case it is possible to obtain the exact function (determination coefficient is equal $R^{2}=0.9998$) of reliability index β(S):

(24) $$ng=6\to \beta \left(S\right)=-0.0023S^{2}-0.0724S+7.7405,\;ng=8\to \beta \left(S\right)=-0.0028S^{2}-0.0889S+7.9446,\;ng=10\to \beta \left(S\right)=-0.0042S^{2}-0.1113S+7.8620,\;ng=12\to \beta \left(S\right)=-0.0063S^{2}-0.1263S+7.7688.$$

## 4. Discussion

Tensegrity structures are currently very popular. However, their analysis is not straightforward due to their initial geometric variability. No studies on the reliability analysis of these structures have been found in the literature known to the authors. The results presented in this article are only the beginning of research on the influence of initial prestressing on the reliability of structures, although it is obvious that the reliability analysis of these structures is particularly important. Furthermore, it should be clearly stated that the original dome (patented by Geiger in 1973) is the best solution. Any modifications do not have a positive effect on the reliability of the structure. It would seem that adding tie rods is safer, but from a reliability point of view, this is not the case.

It has been shown that in tensegrity structures characterized by infinitely small mechanisms, an increase in the level of initial prestressing causes a decrease in the reliability index. In the case of the analyzed Geiger domes, this change is polynomial in nature.

Admittedly, many questions remain unanswered (e.g., what is the impact of non-normal distributions). In future studies, the analysis will be extended to other tensegrity structures, i.e., towers and double-layer grids. Structures with and without infinitesimal mechanisms will be considered.

The intention of this approach was to conduct a conceptual and exploratory study aimed at identifying the fundamental structural and mechanical responses of the system under controlled conditions.

The present findings should be interpreted as preliminary and exploratory, providing a foundation for future investigations involving more realistic loading configurations. In subsequent studies, we plan to extend the analysis to include load combinations derived from design codes. This will allow for a more comprehensive assessment of structural behavior under practical conditions and facilitate direct comparison with engineering applications.

Although the numerical and comparative analyses suggest the original dome exhibits the best performance under the studied static loading conditions, its real-world effectiveness also depends on several key factors:

- Deviations in geometry, material properties, or prestressing levels can significantly influence structural behavior and stability.
- Dynamic and seismic loading introduce additional responses not captured in the present static model.

In tensegrity domes, prestress plays a crucial role in providing structural stiffness and geometric stability, as well as determining the distribution of forces between tensile and compressive members. Asymmetry of prestress leads to uneven force distribution across all elements (local overloads). In practice, asynchronous prestressing is one of the main causes of discrepancies between theoretical models and actual structural performance. Geometric deviations (assembly errors) alter the initial geometry, and even small imperfections can significantly change the response of a tensegrity dome.

Tensegrity domes are typically characterized by relatively low self-weight combined with high flexibility; their natural frequencies are strongly dependent on the level of prestress. In the case of seismic loading, rapid changes in acceleration may cause the dynamic loosening of certain cables. Cyclic loading leads to relaxation and degradation of prestress, which over time reduces stiffness and alters the dynamic characteristics of the structure. For a seismic scenario, static or linear modal analyses may be insufficient; instead, nonlinear time-history analyses and response spectrum analyses are required.

Construction tolerances in prestress and geometric imperfections have a significant, often nonlinear impact on the stiffness, stability, and dynamic response of Geiger tensegrity domes. Dynamic loads (especially seismic ones) can amplify the effects of these tolerances through resonance phenomena, changes in prestress state, and increased vibration amplitudes.

## 5. Conclusions

The main purpose of this paper is to answer the question of which type of design solution of Geiger domes is more sensitive to the impact of the initial prestress on reliability. As stated above, the best solution is regular Geiger dome type B (original dome patented by Geiger). In these cases, the minimum prestress level $S_{min}$ is independent of value of load, position of load, and number of load-bearing girders. Modification of the Geiger patent causes a reduction in the range of variability of prestress level. For some positions and values of load for the minimum prestress level $S_{min}$, the reliability index β is lower the recommended minimum value.

The statement regarding the superiority of the original dome applies within the adopted modeling framework and loading scenario. Future research will extend the analysis to more complex load distributions and structural system representations to verify whether this conclusion remains valid under broader conditions.

Previous studies have shown that the best behavior is achieved by a structure with an initial prestress level close to the effort of structure $W_{max}\approx 0.9$. However, from a reliability point of view, the effort of structure should be lower than $W_{max}\leq 0.6$.

## Figures and Tables

**Table 1:** Relationship between the failure probability $P_{f}$ and reliability index β [59].

Failure Probability $P_{f}$	$10^{-1}$	$10^{-2}$	$10^{-3}$	$10^{-4}$	$10^{-5}$	$10^{-6}$	$10^{-7}$
Reliability Index β	1.282	2.326	3.090	3.719	4.265	4.753	5.199

**Table 2:** Recommended minimum values for reliability index β (ultimate limit states) [59].

Reliability Class	Minimum Values for β	
	1-Year Reference Period	50-Year Reference Period
RC3	5.2	4.3
RC2	4.7	3.8
RC1	4.2	3.3

**Table 3:** Values of self-stress state $y_{S}\;[-]$ for the domes consisting of 6 load-bearing girders.

*S*_1_	*S*_2_	*S*_3_	1	2	3	4	5	6	*C*_1_	*C*_2_	*C*_3_	*C*_4_	*C*_5_	*C*_6_
**Regular** **Geiger Dome Type A**														
−0.380	−0.304	−1.000	0.511	0.368	0.921	0.921	2.006	2.006	-	-	-	0.870	-	1.739
**Regular** **Geiger Dome Type B**														
−0.084	−0.304	−1.000	0.514	0.372	0.921	0.921	2.006	2.006	0.507	0.362	-	0.870	-	1.739
**Modified** **Geiger Dome Type A**														
−0.228	−0.264	−1.000	0.306	0.220	0.801	0.801	2.006	2.006	-	-	0.236	0.756	0.227	1.739
**Modified** **Geiger Dome Type B**														
−0.051	−0.265	−1.000	0.308	0.220	0.801	0.801	2.006	2.006	0.303	0.217	0.236	0.756	0.227	1.739

**Table 4:** Value of the minimum prestress level $S_{min}$ for domes consisting of 6 load-bearing girders [23].

	$S_{min}[kN]$					
	P=1 kN			P=5 kN		
	1 Node	3 Node	5 Node	1 Node	3 Node	5 Node
Regular Type A	2	5	5	8	22	24
Modified Type A	3	8	12	11	34	36
Regular Type B	2	2	2	2	2	2
Modified Type B	14	10	2	41	26	2

## Data Availability

The original contributions presented in this study are included in the article. Further inquiries can be directed to the corresponding author.
